# The Role of Gut Microbiota in High-Fat-Diet-Induced Diabetes: Lessons from Animal Models and Humans

**DOI:** 10.3390/nu15040922

**Published:** 2023-02-12

**Authors:** Yue Qi, Xiaofei Wang

**Affiliations:** National Engineering Technology Research Center for Fruit and Vegetable Processing, Key Open Laboratory of Fruit and Vegetable Processing, Ministry of Agriculture and Rural Affairs, Beijing Key Laboratory of Food Non-thermal Processing, College of Food Science and Nutritional Engineering, China Agricultural University, Beijing 100083, China

**Keywords:** high-fat diet, diabetes, gut microbiota, probiotic

## Abstract

The number of diabetes mellitus patients is increasing rapidly worldwide. Diet and nutrition are strongly believed to play a significant role in the development of diabetes mellitus. However, the specific dietary factors and detailed mechanisms of its development have not been clearly elucidated. Increasing evidence indicates the intestinal microbiota is becoming abundantly apparent in the progression and prevention of insulin resistance in diabetes. Differences in gut microbiota composition, particularly butyrate-producing bacteria, have been observed in preclinical animal models as well as human patients compared to healthy controls. Gut microbiota dysbiosis may disrupt intestinal barrier functions and alter host metabolic pathways, directly or indirectly relating to insulin resistance. In this article, we focus on dietary fat, diabetes, and gut microbiome characterization. The promising probiotic and prebiotic approaches to diabetes, by favorably modifying the composition of the gut microbial community, warrant further investigation through well-designed human clinical studies.

## 1. Introduction

Diabetes, the epidemic of the 21st century, has become one of the major threats to human health and has greatly increased the global burden of the disease [1,2]. The development of diabetes is associated with a number of factors, including excessive dietary intake, genetics, and a sedentary lifestyle [3]. Dietary composition is an important factor influencing the risk of developing diabetes [4], and the quantity and/or quality of dietary fat in diabetes have attracted considerable interest. Dietary fat, especially saturated fatty acid, has been considered to be an unhealthy dietary component due to its high energy density [5,6]. The excessive intake of dietary fat is thought to be associated with obesity and metabolic disorders [7], and the relationship between high-fat diets (HFDs) and diabetes has received extensive attention in past studies. Studies have confirmed that 59% of safflower oil can lead to insulin resistance in rats [8]. With HFD supplementation, beta cell senescence leads to a reduction in insulin release [9].

Gut microbes refer to microbiota present in the gastrointestinal tract and are associated with energy harvesting and storage and the metabolism of many metabolic functions, such as amino acids and carbohydrates [10,11]. Gut microbes are affected by diet, and when mice were shifted to a high-fat, high-sugar diet, the structure of the microbiota was altered within one day [12]. An HFD of 60% lard and soybean oil resulted in a decrease in *Bacteroidetes* and an increase in *Firmicutes* and *Proteobacteria* in mice [13]. Imbalances in gut microbes are associated with metabolic diseases such as obesity and diabetes through mechanisms such as increasing the amount of energy obtained from the diet, affecting fatty acid metabolism in the liver and adipose tissue, and increasing serum concentrations of branched-chain amino acids causing insulin resistance [14,15,16].

This review discusses possible metabolic dysregulation induced by an HFD, particularly the changes in diabetes and gut microbes. In order to mitigate the prevalence of diabetes caused by an HFD, appropriate animal models are selected to explore the cellular and molecular mechanisms between gut microbiome and diabetes. Moreover, this review highlights the anti-diabetic effects of dietary therapy, therapeutic interventions, and probiotics, as well as the mechanisms of their actions.

## 2. High-Fat-Diet-Induced Metabolic Dysfunction

### 2.1. Consumption of Dietary Fats Is Generally Increasing

Over thousands of years, diet consumption, in conjunction with other aspects of daily lifestyles such as exercise, has been associated with metabolic health. As one of the three important nutrients, dietary fats are mainly from edible oils, dairy products, meat, nuts, and other foods. They provide energy, act as a carrier of fat-soluble vitamins, and participate in the metabolism of cells and tissues as biologically active components [17,18]. Inadequate total dietary fat intake can easily lead to malnutrition. However, excessive dietary fat intake is also associated with nutrition-related diseases, including obesity, diabetes, heart disease, and cancer [19].

A typical Western diet contains different forms of fats, such as triglycerides, cholesterol, phospholipids, and long-chain fatty acids. Recently, people’s dietary structure has gradually shifted towards a high-calorie diet with increasing dietary fat intake since the occurrence of economic development and industrialization. The Global Burden of Disease Nutrition and Chronicity Expert Group systematically assessed dietary consumption in 187 countries worldwide and showed that the global average intake of polyunsaturated fatty acids (PUFAs) was about 5%, and the average intake of saturated fatty acids (SFAs) was about 11% [20]. A study of 29 countries found that total trans fat (TFA) intake ranged from 0.3% to 4.2% of total energy intake (E%) in each country, with seven countries having trans-fat intakes above the WHO recommendation of 1% [21]. The Chinese National Nutrition Survey shows that between 1992 and 2002, the total fat intake of Chinese people rose from 22% to 29.8%, with the amount of energy obtained from animal food rising from 9.3% to 13.7% [22]. The excessive intake of dietary fat can lead to the development of various chronic diseases related to fat metabolism.

Both the US Public Health Dietary Recommendations and the UK Public Health Dietary Recommendations state that total fat consumption should be reduced to less than 35% of total energy intake, with the saturated fat intake being limited to less than 10% of daily calories [23]. A high-fat diet in humans refers to a calorie intake of 30–75% [24]. As can be seen from the literature, diets with different higher fatty acid compositions are considered to be HFDs. Stocks T et al. defined an HFD as an intake of 40–45% of energy derived from fat [25]. André J Tremblay defined an HFD in a cohort study as 37% fat intake, with saturated fat intake at 15%, monounsaturated fatty acids (MUFAs) at 12.7%, PUFA at 4.3%, and TFA at 3.5% [26]. Osterberg et al. regarded an HFD as 55% fat intake in their study, with saturated fat accounting for 25% of total energy intake [27]. Cameron J Holloway, in his study, chose an HFD as one in which 70% of the daily calorie intake is fat [28].

### 2.2. Excessive Dietary Fat Intake Exacerbated Metabolic Disorders

Excessive fat intake can lead to excess nutrients in the body and adversely cause systematic metabolic changes in blood plasma, liver, urine, and other organs, involving multiple metabolic pathways, including tricarboxylic acid cycle, glycolysis, lipogenesis, and gut microbiota functions together with the metabolisms of fatty acids, amino acids, choline, and others. These dynamic metabolic responses may result in the development and progression of HFD-induced metabolic disorders, including the dysbiosis of gut microbes [29] and the inflammation of peripheral tissues such as the central nervous system, liver, adipose tissue, and skeletal muscle [30,31].

Although there is still some controversy, a growing body of research points to the development of cardiovascular disease with excessive fat intake (especially SFA) [6,32]. An HFD fed to mice can lead to endothelial dysfunction by reducing the ability of vascular tissue to scavenge superoxide anions [33]. Compared to an HFD with unsaturated fatty acids such as olive oil, a 60%-lard diet reduces endothelial NO synthase activity, thereby affecting vascular homeostasis [34]. In a rabbit model, HFD induction led to early vascular injury through endothelial dysfunction and increased vascular reactivity [35]. A randomized controlled trial (RCT) has also shown that high SFA intake leads to increased plasma concentrations of medium and small LDL particles, increasing the risk of cardiovascular disease [36].

An association between an HFD and cognitive impairment and neurodegenerative diseases has also been found in human epidemiological studies [37]. SFA intake has been positively associated with Alzheimer’s disease, dementia, mild cognitive impairment, and cognitive decline [38,39]. An HFD induces oxidative stress in the brain leading to cognitive impairment and enhances cerebral amyloid angiopathy promoting the development of Alzheimer’s [40,41]. A high-fat palm oil diet for 16 months leads to amyloid deposits in the brains of mice, thought to be a marker of Alzheimer’s disease [42]. Cognitive impairment due to an HFD may be related to oxidative stress. A 60%-lard diet increases brain inflammation in mice, significantly increasing the expression of the cytokines TNFα and IL-6, and the chemokine MCP-1, leading to impaired cognitive performance [40]. In rats, a diet of 40% fat for three months was found to impair learning and memory function, with more severe damage seen with a diet rich in SFA, lard, compared to a diet rich in unsaturated fatty acids, soybean oil [43].

It has been well documented in human and animal models that high-fat diets are associated with fatty liver disease. The HFD diet is widely used to induce hepatic steatosis or non-alcoholic fatty liver disease in experimental animals. The accumulation of triglycerides and cholesterol in the livers of rats fed 35% lard for 12 weeks occurs, which can lead to fatty liver degeneration [44]. Mice fed an HFD developed varying degrees of fatty liver disease [44]. Mice induced with an HFD for 16 weeks showed an obese and inflammatory phenotype, while the liver showed an increase in natural killer T cells and clusters of differentiation (CD)8+ T-cells, which play an important role in obesity-associated adipose tissue inflammation [45]. It has been suggested that total fat intake is positively associated with hepatic steatosis in overweight adolescents [46]. Lisis et al. confirmed that total fat intake was associated with non-alcoholic fatty liver in patients with hepatic steatosis [47].

There is a general consensus that a long-term HFD leads to diabetes, established in both animal and human experiments. Diabetes (defined as fasting blood glucose equal to or above 7 mmol/L) is a chronic metabolic disease caused by insulin abnormalities and manifested as an increase in blood glucose [44,48]. Diabetes can lead to a variety of complications, including retinopathy, nephropathy, peripheral neuropathy, cardiovascular and cerebrovascular complications, arteriopathy of the lower limbs, and hypertension [49]. According to the classification proposed by the American Diabetes Association and adopted by the WHO [45], there are four types of diabetes: type 1 diabetes, type 2 diabetes, gestational diabetes, and other special types of diabetes such as neonatal diabetes, etc. [46]. According to the 10th edition of the Diabetes Atlas published by the International Diabetes Federation [47], the global prevalence of diabetes among people aged 20–79 years was predicted to be about 10.5% (536.6 million people) in 2021, rising to 12.2% (783.2 million people) in 2045. The 2017 Global Burden of Disease Study states that diabetes is the leading cause of diet-related death and disability, second only to cardiovascular disease and cancer [50]. The pathogenesis of type 2 diabetes is complex, and the causes of diabetes have not yet been fully explored. Diabetes is associated with a number of factors, including lifestyle, genetic, and environmental factors, with diet playing an important role in the pathogenesis of type 2 diabetes. Excessive energy intake is thought to be a major cause of the type 2 diabetes epidemic [51]. In a nurses’ health study, higher dietary intake of TFA was associated with increased diabetes [52]. A prospective cohort study noted that whole grain intake was negatively associated with type 2 diabetes [53]. With a long-term HFD, a lack of exercise, genetics, and aging, the body becomes metabolically disturbed, and the balance of blood glucose in the body is disturbed, causing an increase in blood glucose and leading to the development of type 2 diabetes. An HFD is currently one of the main methods of inducing diabetes in rodent models, and HFD-induced diabetes in rodents is associated with weight gain, hyperglycemia, insulin resistance, hyperinsulinemia, and accumulation of lipids [50]. An HFD can lead to hyperglycemia, insulin resistance, and damage to pancreatic beta cells by affecting glucose and lipid metabolism in the metabolic organs [51].

## 3. HFD-Induced Diabetes in Animal Models and Humans

An HFD leads to fat accumulation and increased blood sugar, causing insulin resistance and beta cell damage, causing diabetes in humans [30]. The ethical aspects of human research have necessitated the development of animal models of diabetes. Animal models of diabetes can better explore the pathogenesis of diabetes and help to reveal the pathogenesis of diabetes. Currently, common models of diabetes include rodents, non-human primate models, large animals, and non-mammalian models [54]. Within these models, HFD induction is a common approach, and common symptoms of HFD-induced diabetes in animal models include weight gain, hyperinsulinemia, and disruption of glucose homeostasis [55].

### 3.1. HFD-Induced Diabetes in Human Intervention Studies

The strongest evidence about the relationship between diet and the progression of disease comes from RCTs. Most of the current studies on the relationship between an HFD and diabetes are cohort studies. An HFD can lead to high type 2 diabetes by affecting glucose and lipid metabolism, which in turn can impair the function of major metabolic organs [56], including adipose tissue, pancreas, and liver. Table 1 summarizes the relationship between dietary fat and diabetes in human studies.

Adipose tissue is a loose connective tissue consisting of cells filled with lipids [57]. As an important organ involved in energy homeostasis, adipose tissue produces various bioactive substances, such as adipocytokines and fatty acids, which play a key role in the development of diabetes [58]. An HFD also has an effect on gene expression in adipose tissue; an RCT of patients with metabolic syndrome found that a high saturated fat diet increased the expression of lipolytic genes, which may be associated with impaired insulin sensitivity [59].

The pancreatic beta cells can maintain blood glucose stability by secreting insulin to promote glucose uptake by peripheral tissues [60]. Type 2 diabetes eventually develops when pancreatic beta cells do not secrete enough insulin to meet the demands of insulin resistance. The decrease in beta cell mass in type 2 diabetics is due to beta cell apoptosis [61]. In pre-diabetes, blood glucose can still be maintained at normal levels due to the compensatory response of the beta cells [62]. As oxidative stress and inflammatory responses proceed in later stages, the compensatory mechanisms of the beta cells are continuously compromised, eventually leading to the development of type 2 diabetes [63].

Under normal physiological conditions, hepatic glucose production is regulated by a combination of insulin and glucagon, with glucagon inducing hepatic glucose production and insulin inhibiting it [64]. As there is insulin resistance in diabetes, the inability of insulin to suppress liver glucose production leads to hyperglycemia [65]. An HFD can lead to fat accumulation in the liver, causing insulin resistance and thus disrupting blood glucose homeostasis. An HFD has been shown to significantly increase liver fat levels in 56% of obese women [66].

**Table 1:** High-fat diet and human diabetes intervention studies.

Diet	Participants	Duration	Findings	References
Randomized controlled intervention trials (RCTs)				
50 E % carbohydrate, 20 E % protein, 5 E% PUFAs SFA: 20 E% SFAs, 5 E% MUFAs cis-MUFA: 20 E% cis-MUFAs, 5 E% SFAs trans MUFA: 20 E% trans-MUFAs, 5 E% SFAs	Obese type 2 diabetes patients aged 42–58 (*N* = 16)	6 weeks	No difference in postprandial glucose and serum lipids; increased serum insulin and C-peptide for SAT and trans MUFA diets	[67]
45 E% carbohydrate, 15 E% protein Saturated fat diet (butter and margarine) Monounsaturated fatty acid diets (oleic acid)	Healthy people aged 30–65 (*N* = 162)	3 months	Insulin sensitivity was significantly impaired for SAT diet, while there was no difference for MUFA diet	[68]
Control group: regular diet Intervention group: carbohydrate >50 E%, fat <30 E%	Overweight people aged >40 with glucose tolerance (7.8–11.1) mmol/l (*N* = 102)	3.1 years	55% reduction in the incidence of diabetes in the intervention group	[69]
Cohort				
Fat intake (total, SFA, MUFA, and PUFA)	Healthy people aged 40–69 (*N* = 1173)	2 years	Total fat is negatively associated with insulin sensitivity	[70]
Fat intake (SFA, MUFA, PUFA, TFA, long-chain omega-3 PUFA, and animal and vegetable fat)	Healthy women aged 45–50 (*N* = 35,988)	11 years	Diabetes incidence is negatively associated with vegetable fats	[71]
Fat intake (total fat, SAT, MUFA-oleic acid, PUFA-linoleic acid)	Healthy men aged 40–75 (*N* = 42,504)	12 years	Total fat and SAT intake are associated with a higher risk of type 2 diabetes	[72]
Foods high in fat (vegetable oils, butter, margarine, nuts and seeds, and cakes and biscuits)	European Prospective Investigation into Cancer (*N* = 340,234)	9 years	Margarine consumption is positively associated with diabetes risk	[73]
Fat intake (SFA, MUFA, PUFA, TFA, animal fats, vegetable fats, marine omega-3 fatty acids, non-marine omega-3 fatty acids, and omega-6 linoleic acid (18:2n-6))	The people who were free of diabetes but were at high cardiovascular risk were aged 55–80 (*N* = 3349)	4.3 years	SAT and animal fats (cheese and butter) are associated with a higher risk of diabetes	[74]
Fat intake (SFA, MUFA, and PUFA)	Healthy women aged 45–50 (*N* = 8370)	6 years	Intake of MUFA, total n-3 PUFA, α-linolenic acid, and n-6 PUFA were positively associated with the incidence of diabetes	[75]
Total fat, SFA, MUFA, PUFA, and TFA	Healthy women aged 45–50 (*N* = 84,204)	14 years	TFA intake was positively associated with the risk of diabetes, while PUFA intake was negatively associated with the direction of diabetes	[76]
Type of fat and amount of fat: oils and margarine used during cooking and at the table	Healthy women aged 30–55 (*N* = 83,648)	32 years	Higher intakes of linoleic acid are associated with a lower risk of type 2 diabetes	[77]
	Healthy women aged 25–44 (*N* = 88,610)	22 years		
	Healthy men aged 40–75 (*N* = 41,771)	26 years		
Consumption of nuts and peanut butter (monounsaturated and polyunsaturated fatty acids)	Healthy women aged 35–49 (*N* = 83,818)	16 years	Women who ate nuts or peanut butter at least five times a week had a lower risk of developing diabetes	[78]

### 3.2. HFD-Induced Diabetes in Animal Models

Intervention human studies have necessitated the development of animal models of diabetes, including mice, rats, *Drosophila*, zebrafish, and so on, to better explore the pathogenesis of diabetes and help to reveal the pathogenesis of diabetes. An HFD affects the major insulin-sensitive tissues of the animal model, including adipose tissue, the pancreas, and the liver. Rodent models are the most widely used animal models of diabetes and have been well-studied. Large animal models have physiological conditions more similar to those of humans, including physiological and pathological features. Primate models are very similar to humans but are more expensive and have a longer life cycle. Non-mammalian models, such as fruit flies and zebrafish, have a variety of advantages, such as short growth cycles, simple husbandry, low cost, and high reproductive capacity [54,79]. Table 2 summaries the animal models induced by an HFD, including fat type and amount, duration, animal species, and symptoms

Adipose tissue has an important role in the development of diabetes, and adipose tissue is a major site for storing gluconeogenic substrates and energy [80]. An HFD can alter the expression of genes in adipose tissue, down-regulating genes encoding lipid metabolizing enzymes or markers of lipid differentiation, and increasing genes encoding markers of inflammation [81]. The B-cell activating factor (BAFF) is a tumor necrosis factor (TNF) ligand family protein that is a key factor in the development of poor glucose tolerance [82]. Mice fed an HFD had significantly increased BAFF in visceral adipose tissue and serum [83]. The pro-inflammatory cytokine TNF-α is associated with insulin resistance [84], and an HFD of both lard and soybean oil can increase TNF-α expression levels in adipose tissue [85].

The pancreas is a key site for regulating the secretion of insulin and glucagon, and an HFD can have an impact on the pancreas, leading to the development of diabetes. Several studies have pointed out the mechanism by which an HFD can enhance the compensation of pancreatic β-cells. For example, Jonatan Ahrén et al. found an increase in β-cell volume and β-cell numbers after feeding mice with a 60% lard diet for three months [86]. Kanno et al. found that the compensatory mechanism of islet cells in those on an HFD resulted mainly from increased levels of insulin translation [87]. Ribeiro et al. found that an HFD induced islet hypertrophy and a compensatory morpho-functional shift in pancreatic β-cells [88]. However, it has also been suggested that an HFD can directly lead to the degeneration of islet cell function. The levels of glucose transporters (GLUT)2 and glucokinase mRNA in rat pancreas were significantly reduced after 10 weeks of HFD feeding, and an HFD can reduce insulin secretion by impairing signal transduction in pancreatic β-cells [89]. In ZDF rats fed an HFD for a long period of time, the pancreas developed fat accumulation, which may have led to pancreatic fibrosis, acinar cell damage, and pancreatic stellate cell activation [90,91].

The liver is the main site of carbohydrate and fat utilization and plays an important role in controlling glucose intake, fat metabolism, and energy balance [92]. Liver fat accumulation is associated with insulin resistance, and excess fat intake leads to increased levels of free fatty acids and increased triglyceride deposition in the liver [93].

**Table 2:** Animal model of high-fat-diet-induced diabetes mellitus.

High-Fat Diet	Duration	Mode	Findings	References
335 g/kg corn oil and lard	11 weeks	Japanese fancy mouse 1	Impaired glucose tolerance, hyperglycemia, hyperinsulinemia, and obesity	[94]
58% lard	12 months	C57BL/6J mice	Weight increase, circulating insulin increase, and impaired glucose tolerance	[55]
42% lard 42% olive	12 weeks	Male Wistar rats	Obesity and insulin resistance	[95]
43% fat	Different ages	Nile rat	Hyperinsulinemia, high blood glucose, insulin resistance, abdominal adiposity, and impaired glucose clearance	[96]
20% coconut oil	14 days	*Drosophila*	Induced insulin resistance, elevated triglyceride and circulating glucose, and elevated expression of glass bottom boat (a *Drosophila* homolog of mammalian transforming growth factor-β)	[97]
30% fat vegetable shortening and beef tallow	8 weeks	Guinea pigs	Impaired glucose tolerance, β-cell hyperplasia, compensatory hyperinsulinemia, and dyslipidemia with hepatocellular steatosis	[98]
80% fat (lard)	7 weeks	Dogs	Decreased insulin sensitivity	[99]
8% trans fatty acids	6 years	African green monkeys	Increased intra-abdominal fat deposition, hyperinsulinemia, elevated fructosamine, and reduced muscle AKT (protein kinase) phosphorylation	[100]
Six feeds/day (11% fat)	8 weeks	Zebrafish	Increased blood glucose, impaired glucose tolerance, and insulin resistance	[101]

### 3.3. Gut Microbiota Dysbiosis in HFD-Induced Diabetes

From a physiological point of view, one of the most important links between an HFD and diabetes is the gut microbiota–host axis, as well as the factors released from intestinal metabolites, mediating bidirectional communication between the intestines and the host. Specific intestinal flora community profiles have been suggested to promote type 2 diabetes. Type 2 diabetic patients have dysbiosis of gut microbes with a reduced abundance of butyrate-producing bacteria (including *Eubacterium rectale*, *Roseburia intestinalis*, and *Roseburia inulinivorans*) and an increased abundance of pathogens (such as *Clostridium ramosum*, *Clostridium symbiosum*, *Eggerthella lenta*, and *Escherichia coli*) [102]. Emerging evidence demonstrates that changes in the ratios between gut microbiota, such as the ratios of *Bacteroides* and *Firmicutes*, are associated with the development of type 2 diabetes [103].

Recent studies have also found that type 2 diabetes caused by an HFD may be associated with gut microbes. Diet is a major factor influencing the composition and function of gut microbes. Animal experiments have shown that an HFD affects gut microbes. Compared to the normal diet, mice fed a high-fat diet were more susceptible to diabetes, which may be associated with a reduction in *Bifidobacteria* [104]. In mice on a 45% HFD, there was a decrease in *Bacteroidetes* and an increase in *Firmicutes* and *Proteobacteria* [13]. The HFD reduced the mice’s *Akkermansia*, *Coprococcus*, and *Ruminococcus*, increased *Odoribacter* and *Parabacteroides*, and led to a reduction in short-chain fatty acids (SCFAs) [105]. SCFAs can alleviate diet-induced insulin resistance, and a reduction in SCFAs may lead to type 2 diabetes [106]. An HFD can affect the production of immunoglobulin A, a key regulator of glucose homeostasis, an immune-derived molecule in the gut [107]. Table 3 below summaries the gut microbiota of animals with high-fat-diet-induced diabetes.

## 4. Measurements to Treat Diabetes

With aging and urbanization, the number of people with diabetes is increasing, and the prevalence of diabetes continues to rise [136]. Therefore, the prevention and treatment of diabetes is now an important issue for people. Current treatments for diabetes include medication, dietary interventions, and physical activity [137]. In recent years, with intensive research into gut microbes and diabetes, probiotics may be a new way to treat diabetes.

### 4.1. Therapeutic Interventions for Diabetes

When dietary interventions are not feasible, medication may be considered as a strategy to prevent the development of type 2 diabetics. The chemical drugs used can be divided into biguanides, sulfonylureas, thiazolidinediones, glucosidase inhibitors, etc., according to the mechanism of action and chemical structure [138,139]. Metformin, a biguanide, is the most widely used oral hypoglycemic drug, with the advantages of safety and effectiveness, cardiovascular protection, and low cost, and is recommended as a first-line drug by the American Diabetes Association and the European Diabetes Association [140]. Metformin acts primarily on the liver and can improve hyperglycemia by inhibiting hepatic glucose production [141]. Metformin can act by inhibiting mitochondrial respiratory chain complex I, increasing the AMP/ATP ratio and activating AMPK-activated protein kinase [142,143]. The therapeutic effect of metformin may be related to its effect on gut microbiota, confirmed in animal models and clinical studies. Metformin decreases *Bacteroides fragilis* in type 2 diabetics and also increases the abundance of the mucin-degrading bacterium *Akkermansia* in HFD mice [144,145]. Sulphonylureas are a class of drugs that promote insulin secretion and can act by binding to the SUR subunit of the ATP-sensitive potassium channel in pancreatic cells [146]. However, the effects of sulfonylureas on gut microbiota have still not been well studied. Thiazolidinediones improve insulin sensitivity, and rosiglitazone and pioglitazone are representatives of these drugs. Thiazolidinediones are agonists of peroxisome proliferator receptor gamma (PPARγ), which can enhance insulin target tissues (muscle, fat, liver) and accelerate glucose utilization by activating PPARγ to promote the expression of genes related to glucose transport and lipid metabolism [147,148]. The relative abundance of *Proteobacteria* decreased after the treatment of HFD mice with pioglitazone [149]. Thiazolidinediones may cause a variety of side effects, such as heart failure, cardiovascular death, edema, and fractures [150]. Oral α- glucosidase inhibitors can improve hyperglycemia by delaying the breakdown of carbohydrates into glucose. Currently, the three clinically approved glucosidase inhibitors include acarbose, voglibose, and miglitol [151]. A double-blind RCT of acarbose altered gut microbiota in prediabetic patients, decreasing *Ruminococcaceae* and *Lachnospiraceae* and increasing *Lactobacillaceae*, *Ruminococcaceae*, and *Veillonellaceae* [152].

### 4.2. Dietary Interventions to Alleviate Diabetes

Most health organizations point to dietary interventions as a powerful treatment for diabetes, with controlled diets improving insulin sensitivity and reducing the risk of diabetes and its complications [153]. The American College of Lifestyle Medicine believes that diabetes can be treated with dietary interventions that use whole food, plant-based eating patterns, and increase the intake of unrefined plant foods in the daily diet while eliminating or minimizing the intake of animal foods and refined foods, and with moderate exercise in life [154]. The impact of dietary interventions on diabetes includes effects through indirect weight loss and direct consumption of a variety of nutrients with health benefits [155]. Being overweight is considered to be one of the important factors associated with the risk of diabetes [156,157]. Dietary interventions can reduce weight and improve diabetes by reducing the intake of fat.

As carbohydrate catabolism causes blood glucose to rise, reducing carbohydrate intake in the daily diet can be a good treatment for type 2 diabetes [158]. A low-carbohydrate diet, as defined by the American Diabetes Association, is 130 g/day or less than 26% of total daily energy intake [159]. The traditional Mediterranean diet of minimally processed whole grains has also been shown to have significant benefits for diabetes [160].

Specific types of dietary fat may affect diabetes. The KANWU study found that replacing a diet with monounsaturated fatty acids (23%E for MUFA, 8%E for SFA, 6%E for PUFA) over saturated fatty acids (17%E for SFA, 14%E for MUFA, 6%E for PUFA) improved insulin sensitivity at a total fat intake below the median (37E%) [68]. Another study also confirmed that a diet rich in MUFA could improve central fat distribution and insulin resistance [161]. The Iowa Women’s Study found a reduced risk of diabetes when saturated fatty acids were replaced with unsaturated fatty acids [71], and a study by Summers et al. also noted that replacing saturated fatty acids with unsaturated fatty acids improved insulin sensitivity and abdominal fat accumulation [162]. n-3 PUFA improves high-fat-diet-induced insulin resistance. n-3 PUFA in fish oil improves insulinemia, lipid metabolism, and glucose metabolism in insulin-resistant rats [163]. Different dietary fat types may influence the affinity of insulin receptors by affecting the fatty acid composition of cell membranes [164]. It has also been suggested that dietary fat can modulate the expression of genes involved in lipid metabolisms that affect diabetes, such as fatty acid transport proteins and fatty acid synthases [165]. Increased inflammation may lead to insulin resistance, and PUFA acid intake may improve inflammation, e.g., n-3 PUFA may inhibit Toll-like receptors on the cell surface and reduce the production of inflammatory cytokines [166,167].

Other than the chemical drugs currently used to treat diabetes, natural plant foods such as fruits and vegetables, which are rich in nutrients such as antioxidants and polyphenols, can improve adipokines and oxidative stress, significantly improving beta cell function and insulin sensitivity [168,169]. Table 4 below summaries the potential mechanisms of natural products in food for the treatment of diabetes.

### 4.3. Potential Probiotics Help with Diabetes

Probiotics refer to the beneficial microbiota which inhabit the gut and have a variety of health functions [181]. The development of diabetes is closely linked to gut microbes, and therefore the regulation of gut microbes through probiotics could be a new approach to treating diabetes. The hypoglycemic effect of probiotics has been confirmed in both in vitro and in vivo experiments. Zhu et al. found that most of the Lactobacillus species inhibited dipeptidyl peptidase IV and α-glucosidase by cell-free excretory supernatants and cell-free extracts prepared from 21 Lactobacillus species [182]. Several studies have shown that probiotics can lower blood sugar to varying degrees in diabetic animals, such as *Lactobacillus plantarum* [183], *Lactobacillus casei* [184], *Lactobacillus rhamnosus* [185], and *Clostridium butyricum* [186]. A meta-analysis indicated that probiotics significantly lowered hemoglobin A1c, fasting blood glucose, fasting insulin, triglycerides, and total cholesterol, and improved the symptoms of diabetes [187]. High endotoxemia was demonstrated in high-fat-fed mice. Supplementation with oligofructose to increase the number of gut *Bifidobacteria* revealed that endotoxemia was negatively correlated with *Bifidobacteria* and also improved glucose tolerance and insulin secretion with increased *Bifidobacteria* [188].

The mechanisms by which probiotics improve diabetes include direct effects on the gut microbiota, anti-inflammatory and immunomodulatory effects, reduction in oxidative stress, and involvement in glucose homeostasis [181,189,190]. Gut microbial dysbiosis in diabetic patients leads to increased gut permeability and increased concentrations of bacterial endotoxins such as lipopolysaccharides, inducing inflammation and, ultimately, systemic insulin resistance [191]. Probiotics such as *Lactobacillus paracasei* can restore the expression of the tight junction protein in the colon, thereby reducing serum lipopolysaccharide and inflammatory cytokine levels [192]. Diabetes leads to increased systemic oxidative stress, and the intake of beneficial bacteria can significantly improve fasting blood glucose and the antioxidant status in diabetics [193,194]. *Bifidobacterium lactis* improves glucose uptake and GLU4 translocation through the insulin signaling pathway AKT and insulin receptor substrate-1, increases the expression of GLUT4 and insulin-sensitivity-related genes, and regulates glucose metabolism [195].

## 5. Conclusions

A long-term HFD, especially the excessive intake of saturated fats, could have a variety of adverse effects on human body health and even lead to chronic diseases, including diabetes. Animal models of diabetes can better explore the pathogenesis of diabetes and help to reveal the pathogenesis of diabetes. Daily diet can have a direct impact on the composition and function of host gut microbiota. Excessive fat intake can lead to the imbalances of gut microbiota, including changes in the ratio of *Bacteroidetes* and *Firmicutes*, a decrease in butyrate-producing bacteria, and an increase in the abundance of pathogens. A gut microbiota imbalance may further disturb host metabolism, such as decreased amounts of SCFA and immunoglobulin A, ultimately leading to diabetes. Within animal models, HFD-induced diabetes is accompanied by weight gain, hyperinsulinemia, and the disruption of glucose homeostasis. At present, the treatment of diabetes includes dietary interventions and medication. The causal relationship between gut microbiota and diabetes and its underlying mechanisms are still not fully elucidated, and further research is needed. In the near future, as research into the mechanisms of diabetes and gut microbes intensifies, probiotics may become a new method of treatment for diabetes.

## Figures and Tables

**Table 3:** Effect of an HFD on gut microbes in diabetic animal models.

Mode	Mode	High-Fat Diet	Duration	Sample	Impact on Microbiota	References
Mice	C57BL/6	72% fat (corn oil and lard)	3 months	Ileum, caecum, and colon	Decrease *Bacteroidetes*, *Proteobacteria* Increase *Firmicutes*, *Deferribacteres*, *Lachnospiraceae*	[108]
		60% fat	13 weeks	Caecum	Decrease *Bacteroidetes* Increase *Proteobacteria*	[109]
		60% fat (soybean oil and lard)	8 weeks	Fecal	Decrease *Bacteroidetes* Increase *Firmicutes*, *Deferribacteres*, *Actinobacteria*	[110]
		60% fat	16 weeks	Fecal	Decrease *Actinobacteria* Increase *Proteobacteria*, the ratio of *Bacteroidetes* to *Firmicutes*	[111]
		60% fat +STZ	5 weeks	Fecal	Increased ratio of *Firmicutes* to *Bacteroidetes* Decrease *Rikenellaceae* Increase *Ruminococcaceae* and *Erysipelotrichaceae*	[112]
		60% fat (soybean oil and lard)	18 weeks	Fecal	Decrease *Akkermansia* Increase *Muribaculaceae* and *Eubacterium*	[113]
		60% fat (soybean oil and lard) +STZ	11 weeks	Fecal	Decrease *Bacteroides* Increase *Firmicutes*	[114]
		60% fat (soybean oil and lard) +STZ	6 weeks	Fecal	Increase the ratio of *Firmicutes/Bacteroidetes* Decrease *Akkermansia*, *Muribaculaceae*, *Bacteroides*, *Fusobacterium*, and *Dubosiella* Increase *Colidextribacter* and *Helicobacter*	[115]
	C57BL/6J	60% fat (soybean oil and lard)	8 weeks	Fecal	Decrease *Bacteroidetes* Increase *Firmicutes*, *Proteobacteria*, *Deferribacteres*	[116]
		60% fat (soybean oil and lard)	8 weeks	Cecal	Decrease *Bacteroidetes* Increase *Firmicutes*	[117]
		41% fat	15 weeks	Fecal	Decrease *Akkermansia*, *Coprococcus*, and *Ruminococcus* Increase *Odoribacter* and *Parabacteroides*	[105]
		60% fat +STZ	12 weeks	Fecal	Decrease *Bacteroidetes* Increase *Firmicutes*	[118]
		60% fat (soybean oil and lard)	17 weeks	Fecal	Decrease *Actinobacteria*	[119]
		72% fat (corn oil and lard)	4 weeks	Cecal	Decrease *Lactobacillus* spp., *Bifidobacterium* spp., and *Bacteroides-Prevotella* spp.	[120]
		60% fat (soybean oil and lard)	12 weeks	Fecal	Increased ratio of *Firmicutes* to *Bacteroidetes* Decrease *Bacteroidetes* Increase *Proteobacteria*, *Firmicutes*	[121]
		45% fat (lard)	8 weeks	Fecal	Decrease *Bacteroidetes* and *Actinobacteria* Increase *Firmicutes*	[122]
		60% fat (soybean oil and lard) +STZ	7 weeks	Fecal	Decrease *Verrucomicrobia* Increase *Saccharibacteria*	[123]
		45% fat (soybean oil and lard)	14 weeks	Fecal	Decrease *Akkermansia* Increase the ratio of *Firmicutes* and *Bacteroidetes*	[124]
	BALB/c	40% fat +STZ	8 weeks	Fecal	Decrease *Firmicutes*, *Proteobacteria*, and *Actinobacteria* Increase *Bacteroidetes*, *Actinobacteria*	[125]
	*Swiss*	55% fat	12 weeks	Fecal	Decrease *Firmicutes*, *Actinobacteria* Increase *Bacteroidetes*	[126]
Rats	Wistar rats	58% fat +STZ	12 weeks	Fecal	Decrease *Lactobacillus* spp. Increase *Bifidobacterium* spp.	[127]
		60% fat	6 months	Fecal	Decrease *Actinobacteria*, *Proteobacteria*, and *Bacteroidetes* Increase *Firmicutes*	[128]
	Sprague Dawley rats	60% fat (soybean oil and lard)	10 weeks	Fecal	Decrease *Bacteroides/Prevotella* Increase *Firmicutes*, *Bifidobacterium* spp., *Enterobacteriaceae*, and *C*. *leptum*.	[129]
		10% lard + normal diet	12 weeks	Fecal	Decrease *Firmicutes* Increase *Bacteroidetes*, *Proteobacteria*	[130]
		60% fat (soybean oil and lard)	4 weeks	Fecal	Increase the ratio of *Firmicutes* to *Bacteroidetes*	[131]
		7% lard + normal diet	9 weeks	Fecal	Decrease *Proteobacteria* Increase *Firmicutes*	[132]
		High-fat diet (lard)	12 weeks	Fecal	Decrease *Actinobacteria*, *Proteobacteria* Increase *Firmicutes*	[133]
		High-fat diet (soybean oil and lard)	15 weeks	Colonic	Decrease *Clostridium* and *Faecalibacterium* Increase *Bacteroides*, *Butyricoccus*, *Parabacteroides*, *Rikenella*, *Bifidobacterium*, *Allobaculum*, *Dehalobacterium*, *Lactobacillus*, *Oscillospira*, *Ruminococcus*, and *Desuifovibrio*	[134]
		45% fat (soybean oil)	24 weeks	Fecal and cecal	Decrease *Bacteroidetes* Increase *Firmicutes*	[135]

**Table 4:** Potential mechanisms of natural products in foods in animal models of diabetes.

Natural Products	Model	Potential Mechanisms	References
hesperidin	male C57BL/KsJ-*db/db* mice	↑ hepatic glucokinase activity, glycogen concentration, plasma insulin, C-peptide, and leptin ↓ hepatic glucose-6-phosphatase and phosphoenolpyruvate carboxykinase	[170]
cyanidin 3-glucoside	HFD-induced obese rat and *db*/*db* mice	↑insulin sensitivity, phosphorylation of forkhead box O1 ↓inflammatory cytokines, hepatic triglyceride, c-Jun N-terminal kinase activation	[171]
quercetin	*db/db* mice	↑insulin, triglyceride, glycogen, the ratio of B-cell lymphoma-2/Bcl2-Associated X ↓the activation of caspase-3, -9, -12	[172]
kaempferol	HFD-fed C57BL/6 male mice	↑AKT and hexokinase activity ↓pyruvate carboxylase and glucose-6 phosphatase activity	[173]
ferulic acid	C57BL/KsJ *db/db* mice	↑plasma insulin, hepatic glycogen synthesis, and glucokinase activity ↓total cholesterol and low-density lipoprotein cholesterol	[174]
resveratrol	streptozotocin-nicotinamide-induced diabetic rats	↑insulin ↓blood glucose, glycosylated hemoglobin, TNF-α, IL-1β, IL-6, NF-κB p65 unit, nitric oxide, superoxide dismutase, catalase, glutathione peroxidase, and glutathione-S-transferase	[175]
genistein	streptozotocin-induced diabetic mice	↑insulin, protein expression of cyclin D1, islet β-cell proliferation, survival, and mass	[176]
anthocyanins	HFD-fed Zucker rats	↑adipose and skeletal muscle PPAR activity ↓triglycerides, abdominal fat mass, insulin resistance	[177]
conophylline	streptozotocin-treated and Goto-Kakizaki rats	↑ insulin, β-cell differentiation	[178]
berberine	streptozotocin-induced rats	↑insulin sensitivity, insulin receptor mRNA, protein kinase C activity	[179]
ginsenosides	HFD-fed C57BL/6J mice	↑glucose uptake ↓ TNF-α-induced activation of MAPK and NF-κB signaling pathway	[180]

↑: Increased gene expression, increased content in the body, improved insulin sensitivity. ↓: Gene expression decreases, content decreases.

## Data Availability

Not applicable.
